# Diagnostic performance of gadoxetic acid–enhanced liver MRI versus multidetector CT in the assessment of colorectal liver metastases compared to hepatic resection

**DOI:** 10.1186/s12876-019-1036-7

**Published:** 2019-07-24

**Authors:** Vincenza Granata, Roberta Fusco, Elisabetta de Lutio di Castelguidone, Antonio Avallone, Raffaele Palaia, Paolo Delrio, Fabiana Tatangelo, Gerardo Botti, Roberto Grassi, Francesco Izzo, Antonella Petrillo

**Affiliations:** 1 Radiology Division, Istituto Nazionale Tumori IRCCS Fondazione Pascale – IRCCS di Napoli, Naples, Italy; 2 Gastrointestinal Oncology Division, Istituto Nazionale Tumori IRCCS Fondazione Pascale – IRCCS di Napoli, Naples, Italy; 3 Hepatobiliary Surgical Oncology Division, Istituto Nazionale Tumori IRCCS Fondazione Pascale – IRCCS di Napoli, Naples, Italy; 4 Colorectal Abdominal Surgery Division, Istituto Nazionale Tumori IRCCS Fondazione Pascale – IRCCS di Napoli, Naples, Italy; 5 Pathology Division, Istituto Nazionale Tumori IRCCS Fondazione Pascale – IRCCS di Napoli, Naples, Italy; 6 Radiology Division, Università degli Studi della Campania Luigi Vanvitelli, Naples, Italy

**Keywords:** Liver metastases, Multidetector computed tomography, Magnetic resonance imaging, EOB-Gd-DTPA contrast medium

## Abstract

**Background:**

Imaging is an essential tool in the management of patients with Colorectal cancer (CRC) by helping evaluate number and sites of metastases, determine resectability, assess response to treatment, detect drug toxicities and recurrences. Although multidetector computed tomography (MDCT) is the first tool used for staging and patient’s surveillance, magnetic resonance imaging (MRI) is the most reliable imaging modality that allows to assess liver metastases. Our purpose is to compare the diagnostic performance of gadoxetic acid-(Gd-EOB) enhanced liver MRI and contrast-enhanced MDCT in the detection of liver metastasis from colorectal cancer (mCRC)**.**

**Methods:**

One hundred and twenty-eight patients with pathologically proven mCRC (512 liver metastases) underwent Gd-EOB MRI and MDCT imaging. An additional 46 patients without mCRC were included as control subjects. Three radiologists independently graded the presence of liver nodules on a five-point confidence scale. Sensitivity and specificity for the detection of metastases were calculated. Weighted к values were used to evaluate inter-reader agreement of the confidence scale regarding the presence of the lesion.

**Results:**

MRI detected 489 liver metastases and MDCT 384. In terms of per-lesion sensitivity in the detection of liver metastasis, all three readers had higher diagnostic sensitivity with Gd-EOB MRI than with MDCT (95.5% vs. 72% reader 1; 90% vs. 72% reader 2; 96% vs. 75% reader 3). Each reader showed a statistical significant difference (*p* < <.001 at Chi square test). MR imaging showed a higher performance than MDCT in per-patient detection sensitivity (100% vs. 74.2% [*p* < <.001] reader 1, 98% vs. 73% [*p* < <.001] reader 2, and 100% vs. 78% [*p* < <.001] reader 3). In the control group, MRI and MDCT showed similar per-patient specificity (100% vs. 98% [*p* = 0.31] reader 1, 100% vs. 100% [*p* = 0.92] reader 2, and 100% vs. 96% [*p* = 0.047] reader 3). Inter-reader agreement of lesion detection between the three radiologists was moderate to excellent (k range, 0.56–0.86) for Gd-EOB MRI and substantial to excellent for MDCT (k range, 0.75–0.8).

**Conclusion:**

Gadoxetic acid-enhanced MRI performs significantly better in the detection of mCRC, than MDCT, particularly in patients treated with chemotherapy, in subcapsular lesions, and in peribiliary metastases**.**

## Background

Colorectal cancer (CRC) is the third most frequently detected cancer among both men and women in the United States. Improvements in the therapies of liver metastases, new treatments (e.g., antiepidermal growth factor receptor antibody therapy), and the technological improvements of imaging to increase detection of secondary lesions have mainly prejudiced the survival of patients with distant-stage disease [1]. Imaging is crucial in the management of patients with CRC by detecting the lesions, assessing the number and sites, establishing the resectability, evaluating response to treatment, and detecting drug toxicities and disease recurrences [2–4]. During staging and surveillance it is critical the identification of all lesions, since this is related to a proper patient management. So as, the radiologist, after neoadjuvant chemotherapy, must re-evaluate all lesions detected at first examination to identify responders and non-responders as soon as possible and assess also the lesions that vanished after treatment. In fact, in the era of target therapy, it is recognized that anunseen metastasis in pre surgical diagnostic phase would disastrously reactivate after surgery [5]. Although multidetector computed tomography (MDCT) is the first diagnostic tool used for staging and surveillance, magnetic resonance imaging (MRI) is the only technique that allows evaluating of morphological and functional features that meet these needs [6–8]. Moreover, several liver-specific contrast agent medium have been inserted to improve the hepatic lesions detection and characterization. Gadobenate dimeglumine (Gd-BOPTA) and gadolinium ethoxybenzyl diethylenetriamine pentaacetic acid (Gd-EOB-DTPA) allow obtaining information about the vascularization of the lesions in the different phases of contrast circulation and functional parameters in the delayed, hepatobiliary phase [9, 10].

The aim of this study was to compare diagnostic performance of gadoxetic acid-enhanced liver (Gd-EOB) MRI versus MDCT in the detection of CRC liver metastasis (mCRC), using liver resection as the reference standard.

## Methods

### Study population

Institutional review board of National Cancer Institute of Naples approved this retrospective study. Ethics approval and consent to participate has been waived. From January 2010 to September 2017 we individuated two hundred sixty-height patients with mCRC, who underwent liver resection. Inclusion criteria were the following: *(a)* patients with mCRC pathologically proven; *(b)* with Gd-EOB MRI and liver MDCT examinations within 1 month; *(c)* a period of less than a 1-month between radiological and pathological diagnosis; *(d)* accessibility of resected specimens images. Exclusion criteria were the following: *(a)* discrepancy between radiological and pathological diagnosis, *(b)* poor quality of the resected specimens images and *(c)* absence of Gd-EOB MR or MDCT scans.

**Fig. 1:**
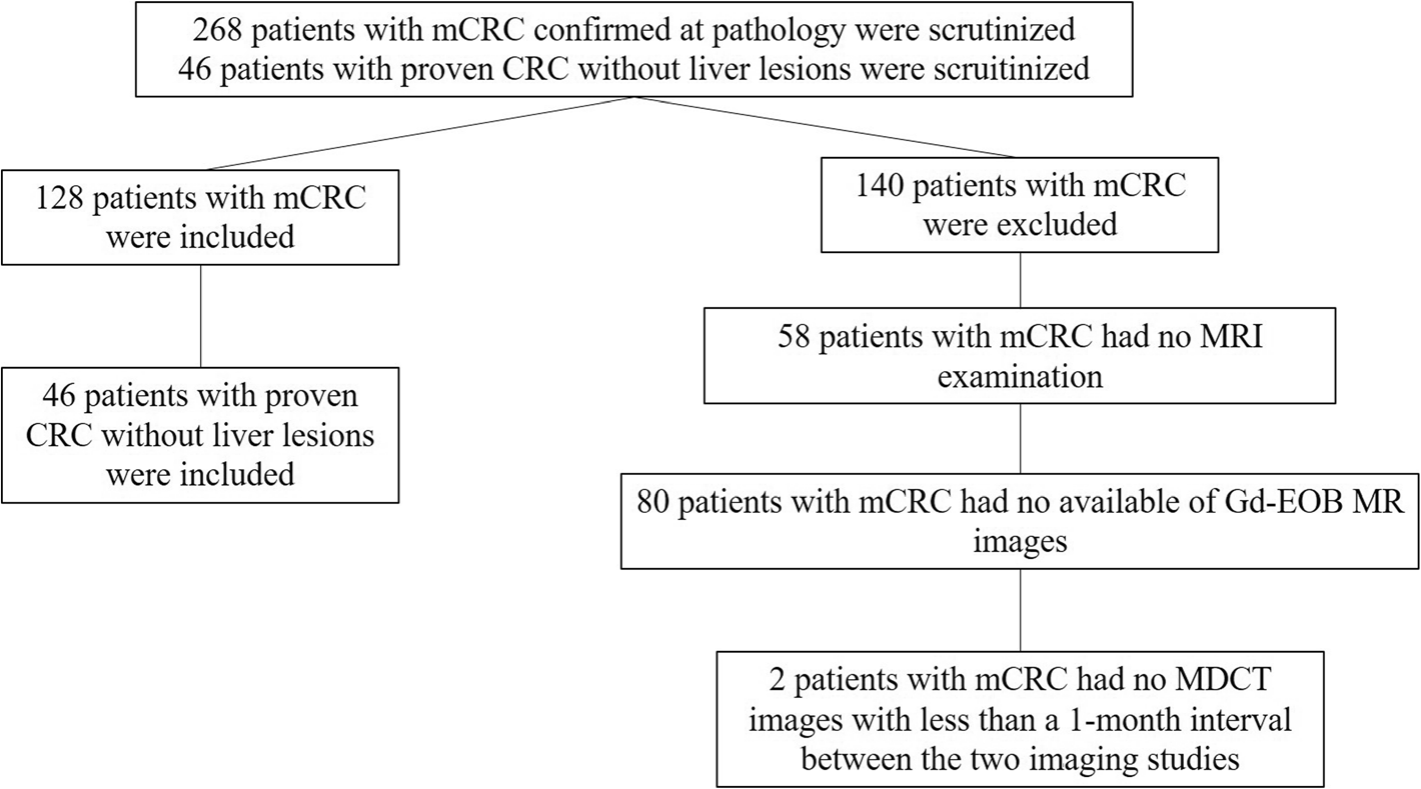
Flow chart for sample selection

Among 268 patients, 68 patients were excluded because MRI examination was not performed. Among 210 selected patients, 82 patients were excluded for the following reasons: *(a)* 80 patients had no Gd-EOB MR scans and *(b)* 2 patients had no MDCT scans less than a 1-month between the two techniques (Fig. 1). Finally, 512 pathologically proven lesions (median 4, range 1–7 per patient), diagnosed as mCRC in 128 patients [72 women-56 men; median age, 58.2 years; range, 33–80 years) comprised our study population. Characteristics of the 128 patients are summarized in Table 1. We also searched the radiological database of our institute during the study period and selected a control group of patients with pathologically proven CRC with subsequent MDCT, Gd-EOB- MR, and ultrasound contrast enhanced study (CEUS); none of the control patients had radiological evidence of liver metastases (as confirmed by at least two techniques) so as to reduce spectrum bias. A total of 46 patients (21 men, 25 women; median age, 56 years [range 33–78 years]) who fit these criteria were enrolled. Characteristics of the 46 patients are also summarized in Table 1.

**Table 1:** Data of the two patients groups

	mCRC patients (no. = 128)	Control patients (no. = 46)	*P* value
Demographics			
Gender	Men 56 (43.7%)	Men 21 (45.6%)	0.82*
	Women 72 (56.2%)	Women 25 (54.3%)	
Age	Median, 58.2 years	Median, 56 years	0.38#
	Range, 33–80 years	Range, 33–78 years	
Primary cancer site			0.001*
Colon	59 (46.1%)	9 (19.5%)	
Rectum	69 (53.9%)	37 (80.4%)	
History of chemotherapy	112 (87.5%)	33 (71.7%)	0.01*
Liver metastases			
Number	512	No one	
	median 4 per patient		
	range 1–7 per patient		
Largest diameter	median 26 mm	–	
	range 12–54 mm		

*Chi square test

# Mann Whitney test

### Lesion confirmation: reference standard

Two pathologists, specialized in the liver, performed histopathologic analysis of resected specimens. One hundred and twenty-eight patients with 512 pathologically proven lesions who underwent surgical resection (median tumor size, 26 mm; range 12–54 mm) constituted the study group. Lesion confirmation was based on the pathologic diagnosis of surgically resected liver specimens. As we previously defined [6], the resected specimens were processed and then sectioned with a 5-mm slice thickness. All tumor samples were labeled with hematoxylin and eosin staining. Immunohistochemical stains were obtained to verify the intestinal origin of the lesions. The group of immunohistochemical markers included cytokeratin 7, cytokeratin 20 and CDX2. The histopathological relationship included increased or infiltrating growth and the presence or absence of tumor sprouting and/or fibrosis and necrosis.

### MDCT protocol

MDCT studies were performed using a scanner with 64 detectors (Optima 660, GE Healthcare, USA). The scan data was 120 kVp, 100–470 mA (NI 16.36), slice thickness 2.5 mm and table speed 0.984 / 1 mm / rotation. The liver protocol included a quadruple phase protocol, counting unenhanced, arterial, portal and equilibrium phases. A non-ionic contrast agent (120 ml of iomeprol, Iomeron 400, Bracco, Milan, Italy) was injected at a rate of 3 ml / s using an automatic power injector (Empower CTA, EZ-EM Inc., New York, USA). The arterial phase was started 19 s after the descending aorta attenuation reached 100 HU, measured by the bolus localization method.

### MR imaging protocol

MR studies was performed with a 1.5 T scanner (Magnetom Symphony, with Total Imaging Matrix Package, Siemens, Erlangen, Germany) with an 8-element body coil and a phased array coil. Liver protocol included the followingsequences: breath hold fat saturated and not fat saturated T2-weighted (T2-w) turbo spin-echo sequence, in- and opposed-phase T1-weighted (T1-w) gradient-echo sequence, dynamic imaging with a fat saturated T1-weighted gradient echo sequence and diffusion weighted imaging. MR imaging parameters for each sequence is summarized in Table 2. Gadoxetic acid (0.025 mmol/kg; Primovist, Bayer Healthcare, Berlin, Germany) was injected intravenously at 1.0 mL/s by a power injector (Spectris Solaris EP; Medrad, Warrendale, Pa). Arterial phase was acquired 7 s after contrast agent arrival at the thoracic aorta using a fluoroscopic monitoring system.

**Table 2:** Pulse sequence parameters on MR studies

Sequence	Orientation	TR/TE/FA (ms/ms/deg.)	AT (min)	Acquisition Matrix	ST/Gap (mm)	FS
Trufisp T2-W	Coronal	4.30/2.15/80	0.46	512 × 512	4 / 0	without
HASTE T2-W	Axial	1500/90/170	0.36	320 × 320	5 / 0	Without and with (SPAIR)
HASTE T2w	Coronal	1500/92/170	0.38	320 × 320	5 / 0	without
In-Out phase T1-W	Axial	160/2.35/70	0.33	256 × 192	5 / 0	without
DWI	Axial	7500/91/90	7	192 × 192	3 / 0	without
Vibe T1-W	Axial	4.80/1.76/12	0.18	320 × 260	3 / 0	with (SPAIR)

Note. *TR* Repetition time, *TE* Echo time, *FA* Flip angle, *AT* Acquisition time, *ST* Slice thickness, *FS* Fat suppression, *SPAIR* Spectral adiabatic inversion recovery

### Images analysis

For each patient, MDCT and gadoxetic acid-enhanced MR studies were independently and blindly assessed in random order within and between three expert radiologists (VG, EdL, AP; 9, 19, and 20 years of experience). The readers were blinded to previous radiological examination, pathologic results and history of previous chemotherapy but were aware that the patients had CRC and thus were at higher risk for developing metastases. To reduce recall bias, all three readers maintained an interval of more than 2 weeks between interpretation sessions of Gd-EOB MR and MDCT images. Each radiologist identified the presence of the metastasis by using the following five-point confidence scale, as we have previously defined [6]: 1 = definitely absent, 2 = probably absent, 3, equivocal, 4 = probably present, 5 = definitely present [11, 12]. the radiologists evaluated the following data: greatest lesion diameter, lesion attenuation at unenhanced CT, signal intensity (SI) on T1- and T2-weighted images, vascular enhancement pattern during arterial, portal, equilibrium phases for CT studies, vascular enhancement pattern during arterial, portal, transitional and HBP phase for MR studies. The SI of the nodule was defined, according to our previous study [6], as isointense, hypointense, and hyperintense compared to surrounding liver parenchyma. We assessed the signal on DWI sequences and measured the ADC of each lesion. The diffusion-weighted signal decay was analyzed using the mono-exponential model, according to the eq. ADC = (ln (S0/Sb))/b, where Sb is the signal intensity with diffusion weighting b and S0 is the non-diffusion-weighted signal intensity. This analysis was region of interest (ROI) based using median value of single voxel signals for each b value. ROIs for the tumor were manually drawn to include such hyperintense voxels on image at b value 800 s/mm^2^. Median diffusion parameters of ROI were used as representative values for each lesion. No motion correction algorithm was used but ROIs were drawn taking care to exclude areas in which movement artifacts or blurring caused voxel misalignments. The enhancement pattern during arterial-, portal-, equilibrium or transitional-, and hepatobiliary phase was described as homogeneous, heterogeneous, peripheral ring enhancement, or target appearance [6]. The latter, due to the central diffusion of contrast medium, was recorded on the hepatobiliary phase images and consisted of a central area of lower degree of hypointensity compared to the periphery of the lesion [6]. In addition, the researchers were asked to record the number and segmental location of the nodule for all detected lesions.

### Statistical analysis

Data were expressed in terms of median value ± range. Chi square test was performed to emphasize significant statistically difference between percentage values in different population subgroups.

Mann Whitney test was performed to relieve statistical difference in median values between two groups. Sensitivities for detection of metastases on per-lesion and per-patient bases and specificity on a per-patient basis were calculated. Lesions that were assigned a grade of 4 or 5 on the confidence scale were regarded as positive for metastases and were considered to be a true-positive finding when lesion presence was pathologically confirmed. We assumed a positive result for per-patient sensitivity if all lesions in a patient were detected. We also assumed a positive result for per-patient specificity when readers correctly assessed patients who did not have any lesion in the radiological results of the control group. Per-lesion detection sensitivities were also assessed according to the pathologic diagnosis and were compared between MDCT and Gd-EOB MR imaging.

Weighted к values were used to evaluate inter-reader agreement of the confidence scale regarding the presence of the lesion. к coefficients in the range of 0.81–1.0 indicated excellent agreement; those in the range of 0.61–0.80, substantial agreement; those in the range of 0.41–0.60, moderate agreement; those in the range of 0.21–0.40, fair agreement; and those in the range of 0.00–0.20, poor agreement.

A *p* value < 0.05 was considered statistically significant. All analyses were performed using Statistics Toolbox of Matlab R2007a (The Math-Works Inc., Natick, USA).

## Results

### Comparison of diagnostic performance between MRI and MDCT in lesion detection

The median interval between MDCT and pathologic confirmation was 15 days. The median interval between MRI and pathologic confirmation was 7 days. We analyzed 512 liver metastases in 128 CRC patients. Lesions size ranged from 12 to 58 mm (median, 28 mm). In terms of per-lesion sensitivity in the detection of metastases, all three readers had higher diagnostic sensitivity with Gd-EOB MRI than with MDCT (95.5% [489 of 512] vs. 72.3% [370 of 512] for reader 1; 89.8% [489 of 512] vs. 71.8% [368 of 512] for reader 2; 96.3% [493 of 512] vs. 75% [384 of 512] for reader 3). Each reader showed a statistical significant difference (*p* < <.001 at chi square test (Table 3). By consensus of three readers, the MRI detected 489 liver metastases while MDCT 384 liver metastases.

**Table 3:** MR and MDCT Performance results for reader

		Sensitivity per lesion	Sensitivity per patient	Specificity per patient
MR	Reader 1	95.5	100.0	100.0
	Reader 2	89.9	97.7	100.0
	Reader 3	96.3	100.0	100.0
MDCT	Reader 1	72.3	74.2	97.8
	Reader 2	71.8	72.6	100.0
	Reader 3	75.0	78.1	95.6

**Fig. 2:**
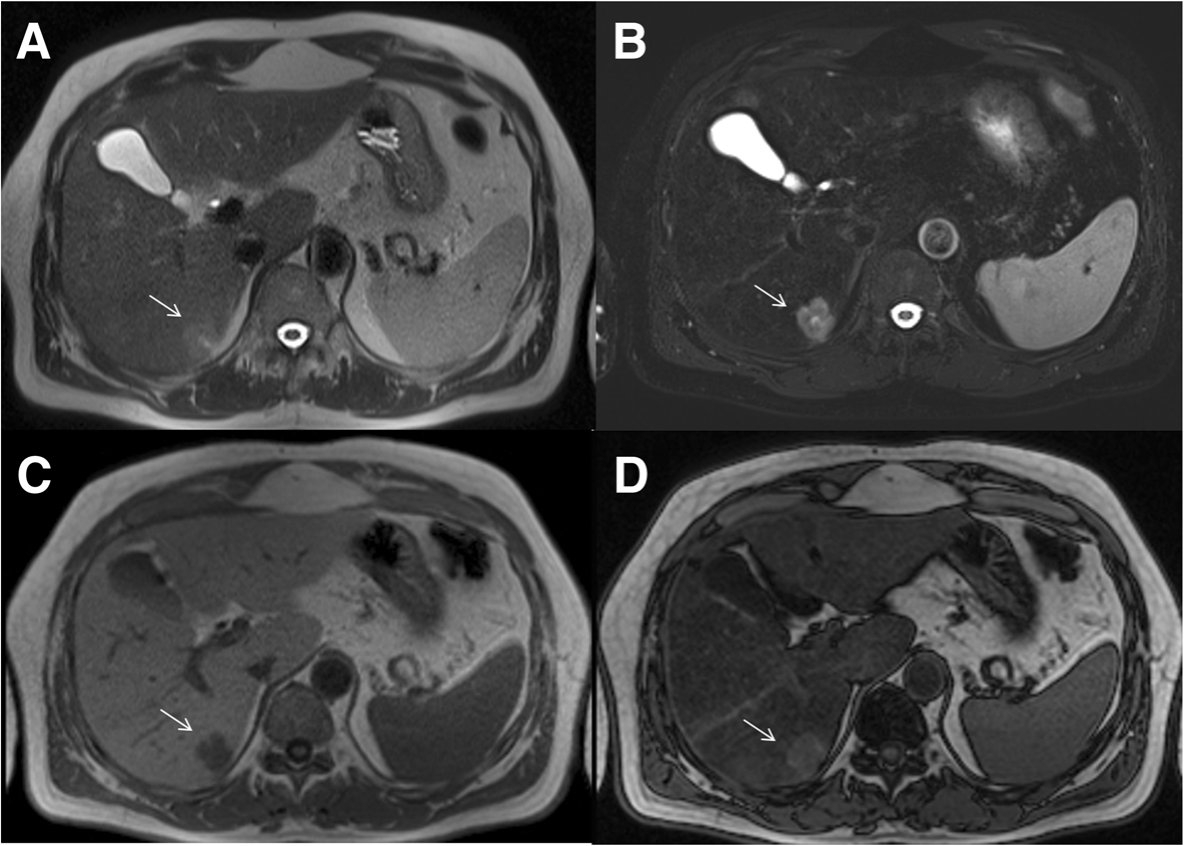
Man 63 year with rectal cancer with hepatic steatosis due to chemotherapy. mCRC on VII hepatic segment. In **a** and **b** (HASTE T2-W and SPACE T2-W FS) the lesion (arrow) is hyperintense with a central area of very high signal intensity. In (**c** and **d**) (T1-W in-out phase sequences) the metastasis (arrow) appears hypointense. In (**d**) it is clear the steatosis

**Fig. 3:**
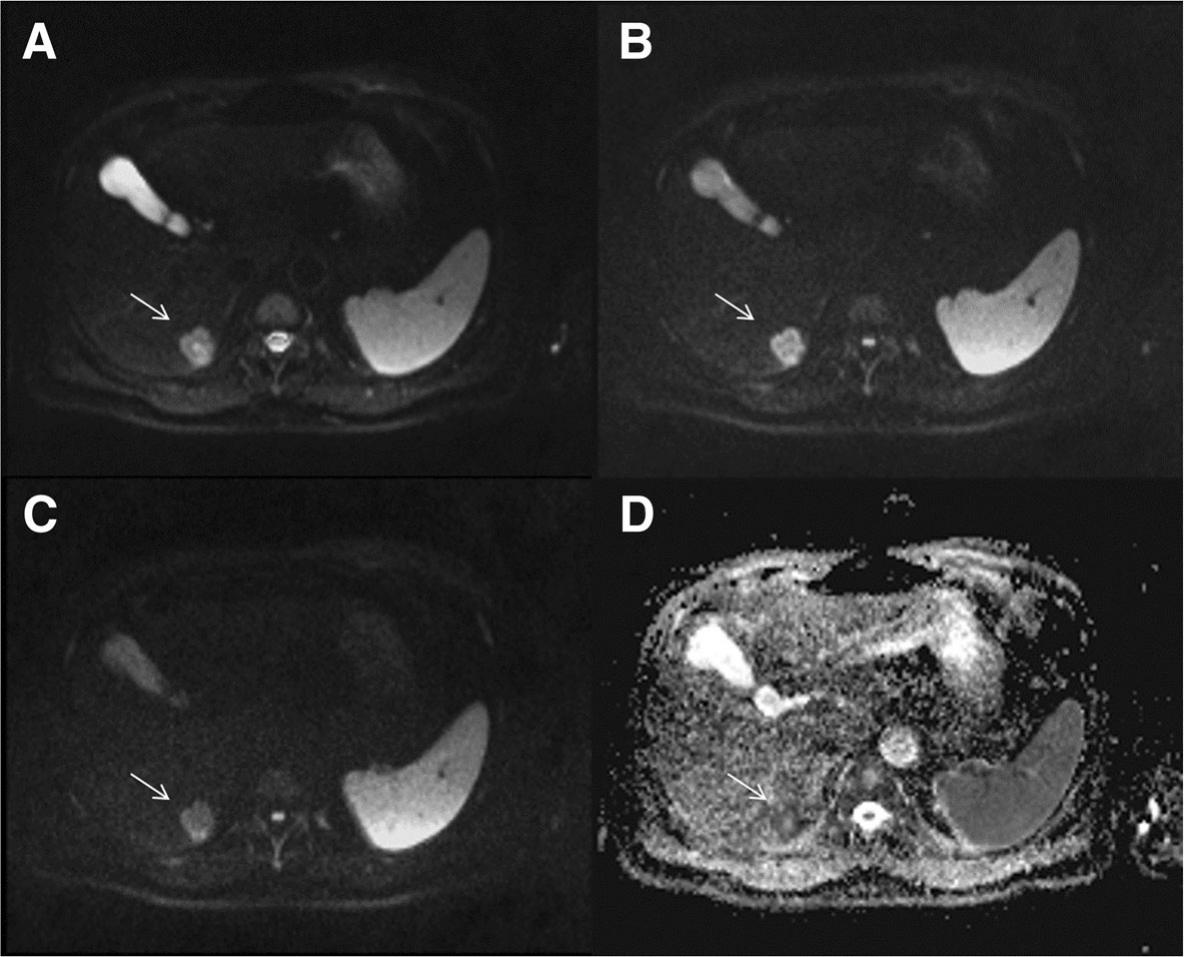
The same patient of Fig. 1. DWI sequences (**a**: b50 s/mm^2^; **b**: b600 s/mm^2^; **c**: b800 s/mm^2^; **d**: ADC map). The lesion (arrow) shows a restricted diffusion for all b values. The ADC value is 1.18 × 10–3 mm^2^/s

**Fig. 4:**
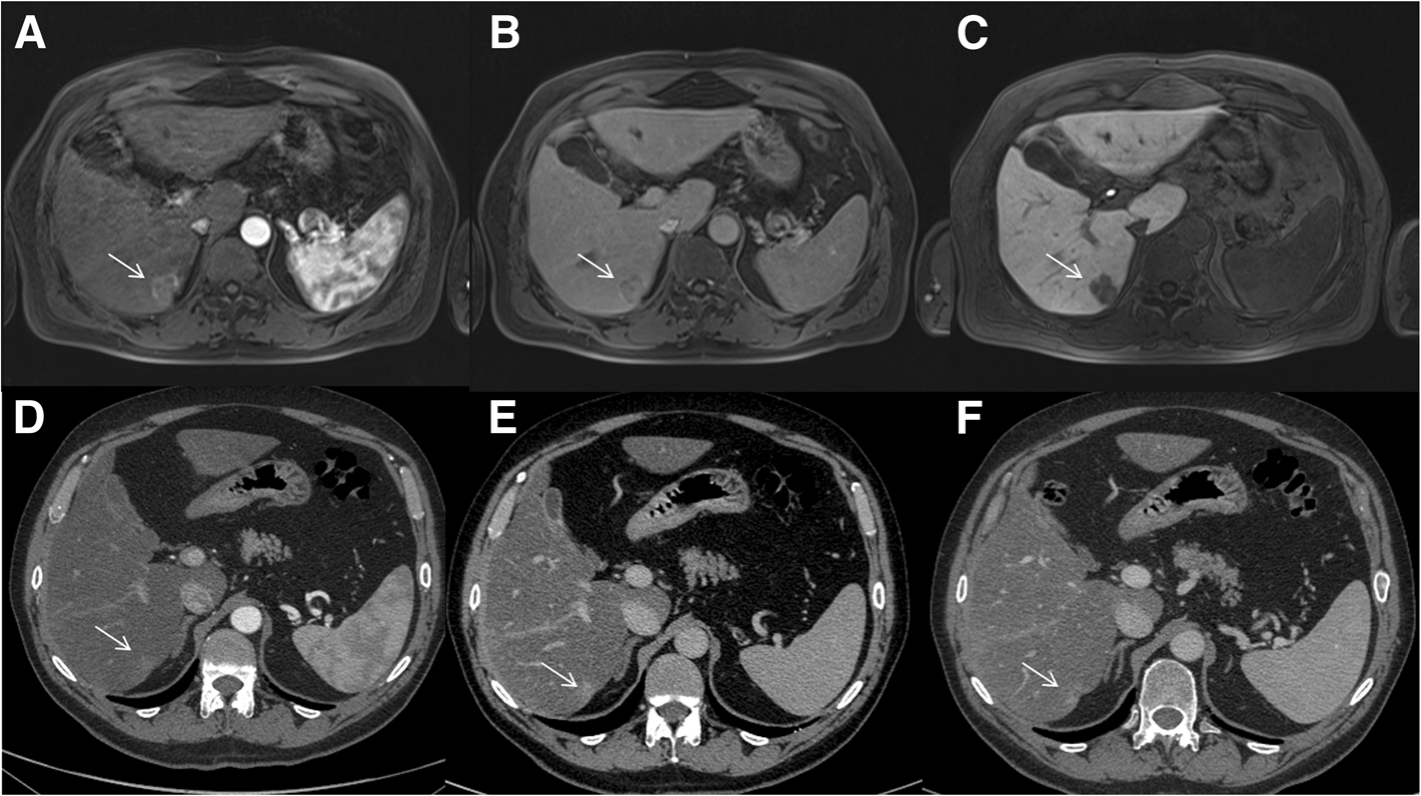
The same patient of Figs. 1 and 2. Contrast study with Gd-EOB-DTPA; the metastasis (arrow) is hypointense with a rim enhancement, in arterial phase (**a**). In the portal phase (**b**), it is hypointense showing an enhancing rim. In the HPB phase, the lesion is hypointense, with a target appearance (**c**). The MDCT contrast study (**d**: arterial phase; **e**: portal phase and **f**: equilibrium phase) does not detected the metastases. There is an inhomogeneous area of hyperattenuation compared to surrounding liver parenchyma

**Fig. 5:**
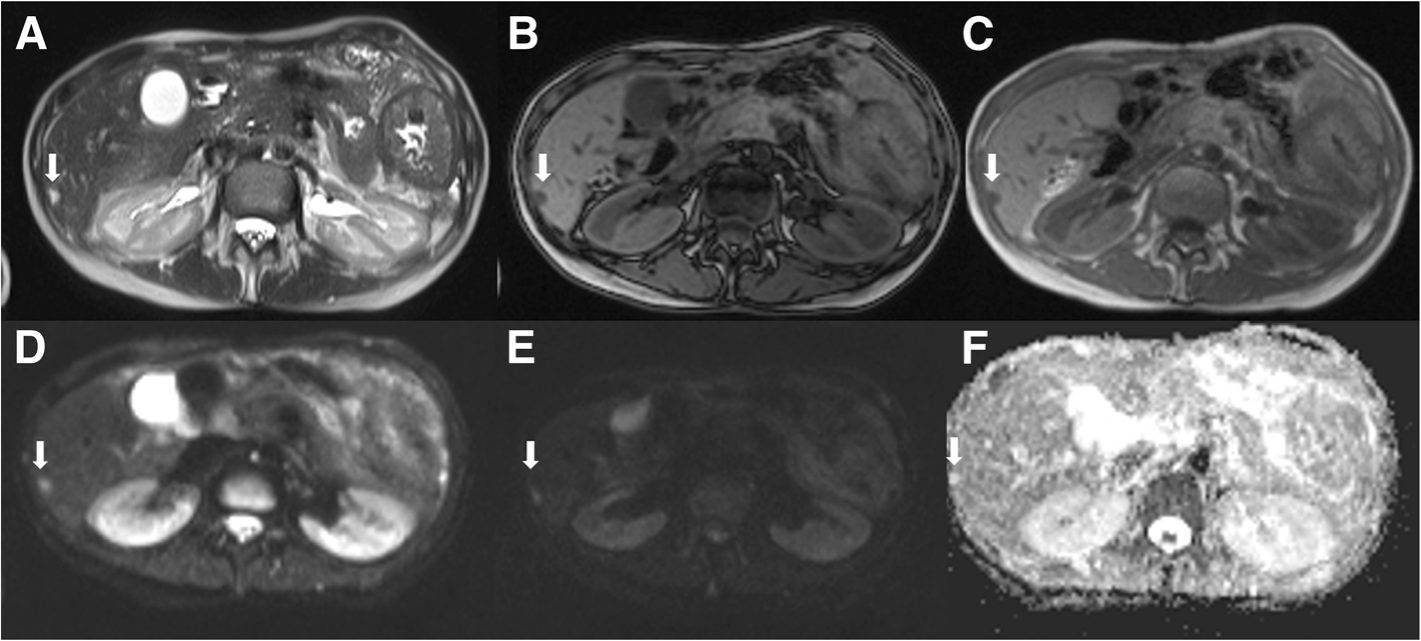
Woman 72 year with colon cancer. Follow up post chemotherapy treatment in presurgical setting. Sub-capsular metastasis (arrow) on VI hepatic segment: in **a** (HASTE T2-W) the lesion appears more hyperintense. In **b** and **c** (T1-W in-out phase sequences) the metastasis (arrow) is hypointense. In DWI sequences (**d**: b50 s/mm^2^; **e**: b800 s/mm^2^; **f**: ADC map), the lesion (arrow) shows a restricted diffusion for all b values. The ADC value is 1.40 × 10–3 mm^2^/s

**Fig. 6:**
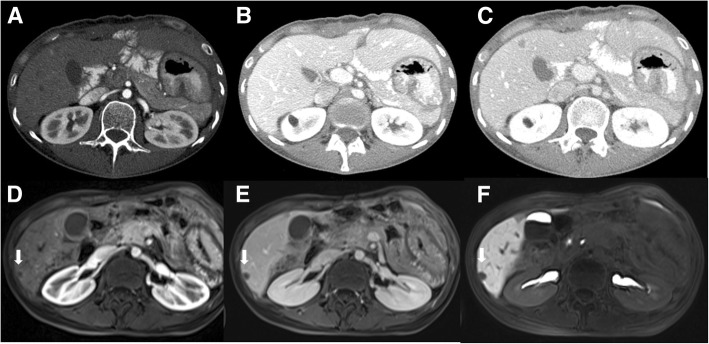
The same patient of Fig. 4. MDCT and Gd-EOB-DTPA contrast study. The MDCT contrast study (**a**: arterial phase; **b**: portal phase and **c**: equilibrium phase) does not detected the metastases. The metastasis (arrow) is hypointense with a rim enhancement, in arterial phase (**d**). In the late phase (**e**), it is hypointense showing an enhancing rim. In the HPB phase, the lesion is hypointense (**f**)

**Fig. 7:**
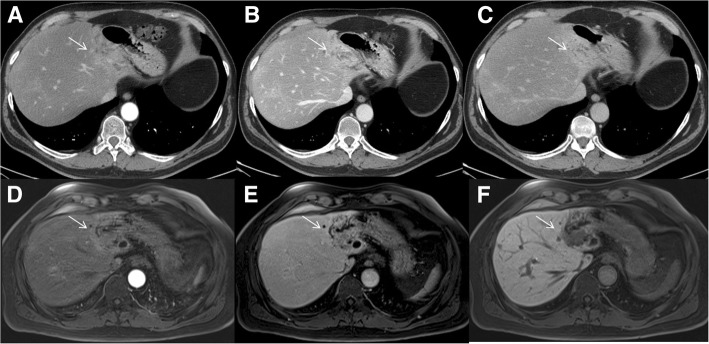
Man 58 year with colon cancer: peribiliary metastasis along left biliary tree. MDCT contrast study (**a**: arterial phase; **b**: portal phase and **c**: equilibrium phase) shows inhomogeneous area of hyperattenuation (arrow) compared to surrounding liver parenchyma with dilation of a biliary branch. Also Gd-EOB-DTPA MR study (**d**: arterial phase; **e**: portal phase) shows inhomogeneous area of hyperintensity (arrow). In HBP phase (**f**) the lesion (arrow) is detected and is hypointense

**Fig. 8:**
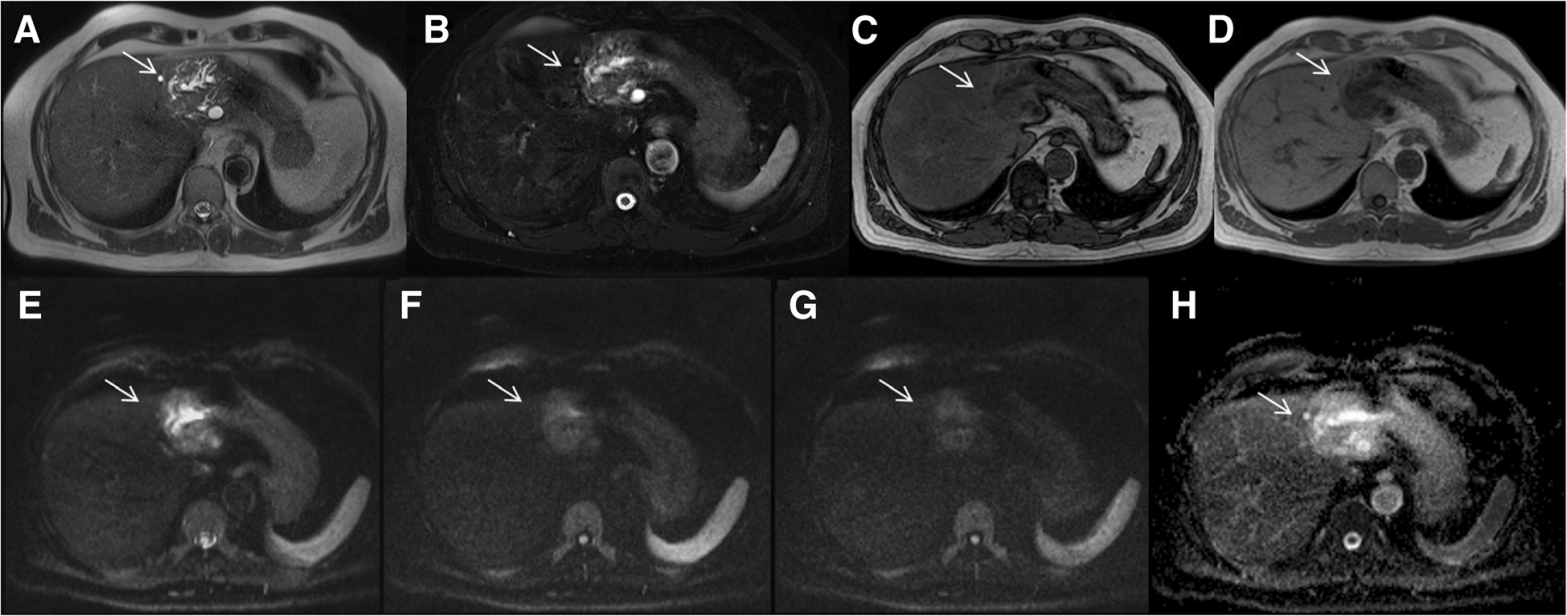
The same patient of Fig. 6. The peribiliary metastasis (arrow) appears hyperintense in T2-W (**a** and **b**; see also the biliary tree dilatation due to the lesion), hypointense in T1-W (**c** and **d**: T1-W out-in phase sequences) with restricted diffusion for all b values in DWI (**e**: b50 s/mm^2^; **f**: b600 s/mm^2^, **g**: b800 s/mm^2^; **h**: ADC map). The ADC value is 1.31 × 10–3 mm^2^/s

The undetected lesions at MDCT were: (*a*) 113 (22.1%) intra-parenchymal metastases (median size 23 mm, range 12–39 mm) in 24 patients (18.7%) (Figs. 2, 3 and 4), (*b*) 23 subcapsular lesions (4.5%; largest diameter 16 mm (range 12–22 mm)) in 3 patients (2.3%) (Figs. 5 and 6), and (*c*) 8 peribiliary metastases (1.6%) in 8 patients (6.25%) (Figs. 7 and 8). The undetected lesions at MRI were: (*a*) 19 sub-capsular lesions (3.7%; (median diameter 14 mm; range 12–15 mm)) in 3 patients (2.3%).

MRI showed higher performance than MDCT in per-patient detection sensitivity, (100% [128 of 128] vs. 74.2% [95 of 128] [*p* < < .001] for reader 1, 97.7% [125 of 128] vs. 72.6% [93 of 128] [*p* < < .001] for reader 2, and 100% [128 of 128] vs. 78.1% [100 of 128] [*p* < < .001] for reader 3), with a statistical significant difference (Table 3).

In the control group, MRI and MDCT showed similar per-patient specificity (100% [46 of 46] vs. 97.8% [45 of 46] [*p* = .31] for reader 1, 100% [46 of 46] vs. 100% [46 of 46] [*p* = .92] for reader 2, and 100% [46 of 46] vs. 95.6% [44 of 46] [*p* = .047] for reader 3) (Table 3).

A false-positive diagnosis in the control group was made at MDCT analysis for one patient for reader 1 and for two patients for reader 3. One lesion detected as metastasis in one patient by reader 1 on MDCT was confirmed to be an area of steatosis at MR study and CEUS. Reader 3 detected the two lesions in one patient, as metastases on MDCT, which were confirmed to be oxaliplatin lesions by MRI study and CEUS, and one lesion in one patient as metastasis on MDCT that was confirmed to be a hepatic adenoma.

Inter-reader agreement of lesion detection between the three radiologists was moderate to excellent (k range, 0.56–0.86) for Gd-EOB MRI and was substantial to excellent for MDCT (k range, 0.75–0.8).

Imaging Features at MRI and MDCT were synthesized in Table 4.

**Table 4:** Imaging Features at MRI and MDCT

		Characteristic	Lesions number (%)
MR	T1-weighed sequences	homogeneously hypointense	489 (100.0%)
	T2-weighed sequences	central area of very high signal intensity	350 (71.5%)
		homogeneous hyperintense	139 (28.4%)
	DWI	restricted diffusion	489 (100.0%) median ADC 1.23 × 10–3 mm2/s
	Gd-EOB MR Arterial phase	rim enhancement	187 (38.2%)
		hypointense	302 (61.7%)
	Gd-EOB MR Portal phase	homogeneously hypointense	406 (83.0%)
		enhancing rim	83 (16.9%)
	Gd-EOB MR transitional phase	low signal intensity compared to the surrounding parenchyma	489 (100%)
	HPB phase	homogeneously hypointense	297 (60.7%)
		target appearance	192 (39.3%)
MDCT	Unenhanced images	isoattenuating	384 (100.0%)
	Arterial phase	isoattenuating	201 (52.4%)
	Portal and equilibrium phase	hypoattenuating	183 (47.6%)
		hypoattenuating	384 (100.0%)

## Discussion

We analyzed the diagnostic performance of Gd-EOB MR and MDCT in detection of metastases in 128 CRC patients showed that the performance was significantly higher with Gd-EOB MR imaging than with MDCT. Also, we showed that all three readers had significantly higher sensitivity in detecting metastases with Gd-EOB MRI than with MDCT. MR imaging also showed higher performance than MDCT in per-patient detection sensitivity. In the control group, MR imaging and MDCT showed similar per-patient specificity.

Gd-EOB MR did not detect 19 sub-capsular lesions while MDCT did not detect 23 sub-capsular lesions, 113 intra-parenchymal metastases and 8 peribiliary metastases. We think that the reason of lower diagnostic performance of MRI in detecting of sub-capsular lesions is the extra-parenchymal lesion side so that the Gd-EOB contrast not improves the detection of lesions by increasing the lesion-to-liver contrast gradient. In addition, these metastases were all small (median value of largest diameter was 14 mm; range 12–15 mm) so that the sequences employed did not allow to detection. Conversely, MRI detected 4 sub-capsular lesions, thanks to T2-W and DWI sequences, that MDCT did not evaluated, since the median value of largest diameter was 20 mm (range 18–22 mm). MDCT has showed lower diagnostic performance for all sub-capsular lesions; also in this case we think that the localization of the lesion effect on lesion-to-liver contrast probably due to progressive enhancement of these metastases.

MDCT has showed a lower diagnostic performance compared to Gd-EOB MRI in detection of peribiliary metastases. As we showed in our previous study all MDCT contrast phases showed a low diagnostic performance in detection of peribiliary metastases and it is related to the typical progressive contrast enhancement of these lesions. Consequently, small lesions may go undetected because of the attenuation similar to that of the surrounding parenchyma [3]. MRI detected all metastases with the best diagnostic performance showed by T2-w and DWI sequences [3]. Although the peribiliary metastases are usually considered as infrequent [13, 14], the presence of these metastases profoundly changes the managing of the patient, excluding a radical surgery when the lesion involves both biliary systems [3].

In our study, MDCT did not detect 113 intra-parenchymal metastases (median tumor size 23 mm, range 12–39 mm) in 24 patients, which underwent previous chemotherapy. Systemic therapy administrated to make resectable a liver metastases is acknowledged as conversion therapy [2]. However, despite the known advantage of neoadjuvant chemotherapy, there are several complications related to of chemotherapy that should be assessed with imaging since they have an impact on surgical morbidity. The chemotherapy-related complications, steatosis, chemotherapy-associated steatohepatitis and sinusoidal obstruction syndrome, might damage the liver, thus influencing the outcome post resection. In addition, liver steatosis decreases the contrast between hepatic parenchyma and metastases, influencing the detection of hepatic lesions [15, 16]. Although, the MDCT is suggested by most clinical trials as the imaging modality to choose in the assessment after neoadjuvant therapy. This is due to its widespread accessibility, standardization, and capability to assess the whole abdomen and chest in one setting [17]. However, there are several features to be evaluated, considering that evidence on the performance of the different imaging techniques for preoperative, after neoadjuvant therapy, imaging of mCRC is inadequate and equivocal and, maybe MDCT is not should be the diagnostic tool to choose [18]. In fact, according to us, several studies have shown that Gd-EOB MRI is more sensitive than MDCT and Positron Emission Tomography (PET)/CT in the detection of the completely treated liver metastases [18]. A meta-analysis of 11 studies [18] that included 906 liver lesions showed that MRI is the most accurate technique after neoadjuvant chemotherapy, with a sensitivity of 86%. MDCT is the best alternative with a sensitivity of 70%. Both PET and PET/CT have instead a poor diagnostic accuracy in the neoadjuvant scenery [18].

In our study we found that the largest diameter of lesion not affect the diagnostic accuracy of two techniques, instead liver steatosis, post chemotherapy, by decreasing the difference in contrast between hepatic parenchyma and lesions, reduces MDCT diagnostic performance. Also Scharitzer et al. compared the diagnostic performance of CT and Gd-EOB-DTPA MRI in mCRC patients [19]. They evaluated metastases < 10 mm and lesions in patients with and without steatosis, showing that, in patients with hepatic steatosis, MRI had a better performance than CT for the assessment of small colorectal liver metastases, although the difference was not statistically significant [19], conversely to us. Instead, according to us, Berger-Kulemann et al. demonstrated that not only Gd-EOB MRI is superior to 64-detector row CT in detecting CRC liver metastases ≤10 mm but also that there was a better performance in patients with liver steatosis [20]. Conversely, Kulemann evaluated the accuracy of CT versus MRI for mCRC and showed no significant difference between the two modalities in the detection of lesions > 10 mm (CT detected 33/51 lesions (65%); MRI 45/51 (88%)), while MRI is superior to CT, for the detection of small lesions (≤10 mm; CT detected only 2/18 (11%) and MRI 12/18 (66%)) [21].

Sofue et al. evaluated the diagnostic performance in the detection of mCRC between 64-detector-row CT alone and the combination of CT and Gd-EOB MRI to assess whether the association of two techniques changing the operative strategy. The study showed as the combination of CT and MRI provided better diagnostic performance than CT alone with a change in operative strategy in one-third of the patients [22].

Although our study reported data well known, related to higher diagnostic performance of Gd-EOB- MRI study compared to MDCT, we assessed several features that we not found in previous studies. First that the diagnostic performance both MRI and MDCT is low in the assessment of subcapsular lesions, and it is not completely related to the largest lesion diameter. In fact, the lower diagnostic performance is due to the extra-parenchymal side, so that the employment of hepatospecific contrast do not increase the lesion to liver contrast. Also the typical progressive contrast of these lesions is another feature that reduce the accuracy of contrast study in detection. Conversely the possibility to use sequences as T2-w and DWI that show high soft tissue contrast resolution, allows an accurate tumor detection. This scenario is similar to that of peribiliary metastases. In fact we are the first group that reported a so large group of patients with peribiliary lesions so that we showed not only imaging features of these but also suggested as these should be evaluated. In patients with small peribiliary metastasis MDCT cannot assessed the lesion so as the indirect signs, while T2-W and DWI sequences can detect it. In this setting, also, EOB-GD contrast not allow to increase the diagnostic performance since the lesion is extraparenchimal. At the end, we evaluated the diagnostic performance of MDCT and MRI in assessment of conversion therapy, showing that as liver steatosis post chemotherapy, by decreasing the difference in contrast between hepatic parenchyma and lesions, reduces MDCT diagnostic performance. In fact, in our study MDCT did not detected 113 (22.1%) intra-parenchymal metastases (median size 23 mm, range 12–39 mm) in 24 patients (18.7%). Therefore, in this setting we think that EOB-Gd MRI is the most reliable imaging modality. Therefore, we think that it would be important that other authors evaluate the role of MDCT and Gd EOB MRI in the assessment of subcapsular and peribiliary lesions and the effects of conversion therapy on diagnostic accuracy of MDCT, in order to support our current results.

Some limitations of our study must be considered. First, as this was a retrospective study, there may have been potential selection bias. Second, Gd-EOB MRI and MDCT studies were performed by different radiologists; however, we made our best effort to use appropriate images with good quality to evaluate all lesions.

## Conclusion

Imaging plays an important role in the workup of patients with CRC. Gd-EOB MRI performs significantly better in the detection of mCRC, especially post chemotherapy, for subcapsular lesions and for peribiliary metastases, than did MDCT. However the diagnostic performance both MRI and MDCT is low in the assessment of subcapsular lesions, and it is not completely related to the largest lesion diameter. In the assessment of peribiliary lesions, so as of subcapsular metastases, T2-W and DWI allow to detect more lesions than EOB-GD contrast. In the assessment of conversion therapy, liver steatosis, decreasing the difference in contrast between hepatic parenchyma and lesions, reduces MDCT diagnostic performance compared to Gd-EOB MRI.

## Data Availability

The datasets generated and/or analysed during the current study are not publicly available but are available from the corresponding author on reasonable request.

## Abbreviations

ADC: Apparent diffusion coefficient
CEUS: Contrast enhanced ultrasound
CRC: Colorectal cancer
DWI: Diffusion weighted imaging
Gd-BOPTA: Gadobenate dimeglumine
Gd-EOB-DTPA: Gadolinium ethoxybenzyl diethylenetriamine pentaacetic acid
HBP: Hepatobiliary phase
mCRC: Metastatic colorectal cancer
MDCT: Multidetector computed tomography
MRI: MAGNETIC Resonance Imaging
PET: Positron emission tomography
ROI: Region of interest
T1-w: T1-weighted
T2-w: T2-weighted
